# Morbidity of School Children in Panauti Municipality of Kavrepalanchowk: A Descriptive Cross-sectional Study

**DOI:** 10.31729/jnma.4825

**Published:** 2020-04-30

**Authors:** Naresh Manandhar, Pratiksha Pathak, Prasanna Lama, Sunil Kumar Joshi

**Affiliations:** 1 Department of Community Medicine, Kathmandu Medical College, Sinamangal, Kathmandu, Nepal

**Keywords:** *body mass index*, *dental caries*, *morbidity*, *nutritional status*, *underweight*

## Abstract

**Introduction:**

School health has been considered as a high priority intervention in developing countries but it has not been prioritized in Nepal. The objectives of the study are to find out the prevalence of morbidity and nutritional status in school children.

**Methods:**

A descriptive cross-sectional study was conducted in a private school located at Panauti Municipality of Kavrepalanchowk district for one week of November 2019. A convenient sampling technique was used. From a selected school, a total number of 356 students studying from Grade I to X were included in the study using. Confidence Interval at 95% was calculated along with frequency and proportion for binary data using Statistical Package for Social Science (SPSS) software.

**Results:**

Among 356 students, the most common three morbidities were dental caries 43 (18.1%), tonsillitis 26 (6.2 %), and headache 18 (7.1 %). Based on weight for age, 43 (23.9%) boys and 22 (12.5%) girls were underweight and 12 (6.7%) boys and 4 (2.7%) girls were overweight and 6 (2%) were obese. Thus school health programs should give more emphasis on oral health, nutrition, personal hygiene, etc.

**Conclusions:**

The health and nutritional status of school children in this study were found to be satisfactory compared to other studies. The present study emphasized oral health. The school health program is important in the school for the prevention of diseases like a parasitic infestation, improving personal hygiene, and nutritional status.

## INTRODUCTION

World Health Organization's Global School Health Initiative, seeks to mobilize and strengthen health promotion and education activities at the local, national, regional and global levels. The Initiative is designed to improve the health of students, school personnel, families and other members of the community through schools.^1^ In 1997, WHO developed 10 recommendations for school health, and initiated a global school health initiative in ten countries, of which 8 were developing countries.^1^ School health program has included essential components of public health for cost-effective health program, the nutrition, and health of school children. Nepal's poverty was 21% in 2018.^2^ Poor people are more likely to be affected by different forms of malnutrition. Malnutrition increases health care costs, reduces productivity and slows economic growth, which can perpetuate a cycle of poverty and ill health.^3^

The objectives of the study are to find out the prevalence of morbidity and nutritional status in school children.

## METHODS

A descriptive cross-sectional study was conducted in a school of Panauti Municipality, Kavrepalanchowk district for 10th to 15th November 2019. Ethical approval was taken from the Institutional Review Committee of Kathmandu Medical College. The purpose of the study was explained to the school Principal and permission was taken from the Principal and parents of students to conduct the study. Students studying from grade I to X were taken into the study. Those who were absent or were not willing to participate were excluded from the study.

The structured questionnaire was developed. The questionnaire consisted of demographic information, physical and clinical examination. The instruments used were a weighing machine and measuring tape. Each child's weight was measured in kilograms. The scale was calibrated against known weight regularly. Zero error was checked for and removed every day if present. Students wore only light clothes while measuring weight in the classroom. The weights were recorded to the nearest 100g. Height in centimeters was marked on the wall in school with the help of a measuring tape. The students were asked to remove their shoes and stand with heels together and head positioned in such a way that the line of vision was perpendicular to the body. A scale was brought down to the topmost point on the head of students standing against the wall where the calibration was done. The height was measured to the nearest 0.5cm. Health Examination of all students underwent a thorough physical and systemic examination including medical history. The Convenient sampling technique was used. Selection bias and measurement bias has been addressed as far as possible.

Collected data were entered into Microsoft Excel. Data analysis was done using SPSS version 20. Frequency and percentage were calculated. BMI for age was computed using Group BMI Calculator, Metric, v1.0-CDC.

## RESULTS

Out of 356 students, the morbidity was present in 101 (28.37%) school children, commonest was Dental caries 42 (11.08 %), and followed by Tonsillitis 26 (7.3%). Headache and Eye problems were found to be 18 (5.06%) and 17 (4.78%) school children respectively. The morbidities were found higher in boys compared to girls except for ear problems (Table 1).

**Table 1:** Morbidities in boys and girls except ear problems.

S.N.	Morbidities	Boys n (%)	Girls n (%)	Total n (%)
1	Dental caries	22 (12.22)	21 (11.93)	43 (12.08)
2	Tonsillitis	14 (7.78)	12 (6.82)	26 (7.30)
3	Headache	10 (5.56)	8 (4.55)	18 (5.06)
4	Refractive error	10 (5.56)	7 (3.98)	17 (4.78)
5	Ear problem	2 (1.11)	6 (3.41)	8 (2.25)
6	Skin problem	4 (2.22)	2 (1.14)	6 (1.69)
7	Gastritis	3 (1.67)	1 (0.57)	4 (1.13)
8	Abdomen pain	-	4 (2.28)	4 (1.13)

There were 180 (50.6%) boys and 176 (49.4%) girls included in the study. The mean age of students was 11.38±2.83 years. The age of the students ranged from 6 to 17 years (Table 2). The majority of them were between the ages of 11 to 15 years.

**Table 2:** Distribution of school-age children by age and sex.

Age (years)	Sex		Total
	Boys n (%)	Girls n (%)	n (%)
**6**	9 (50.0)	9 (50.0)	18 (5.1)
**7**	15 (51.7)	14 (48.3)	29 (8.1)
**8**	10 (55.6)	8 (44.4)	18 (5.1)
**9**	12 (38.7)	19 (61.3)	31 (8.7)
**10**	25 (73.5)	9 (26.5)	34 (9.6)
**11**	19 (46.3)	22 (53.7)	41 (11.5)
**12**	17 (37.8)	28 (62.2)	45 (12.6)
**13**	31 (60.8)	20 (39.2)	51 (14.3)
**14**	18 (47.4)	20 (52.6)	38 (10.7)
**15**	11 (37.9)	18 (62.1)	29 (8.1)
**16**	9 (52.9)	8 (47.1)	17 (4.8)
**17**	4 (80.0)	1 (20.0)	5 (1.4)
**Total**	180 (50.6)	176 (49.4)	356 (100.0)

The mean and standard deviation of height, weight, and Body Mass Index (BMI) were 1.40m±0.16m, 33.82 kg±12.01kg and 16.53 kg/m2 ±2.98 kg/m2 respectively. The proportion of school children with normal BMI was 269 (76%), out of which 124 (68.9%) were boys and 145 (82.4%) were girls. Sixteen (4.5%) of the students were overweight, 12 (6.7%) were boys. Under the obese category, there were 6 (1.7%) students in which 5 (2.8%) were boys (Table 3).

**Table 3:** School children’s BMI-for-age.

Label	Boys n (%)	Girls n (%)	Total n (%)
Underweight	43 (23.9)	22 (12.5)	65 (18.3)
Normal BMI (5th - 85th Percentile)	124 (68.9)	145 (82.4)	269 (75.6)
Overweight or obese (≥85th Percentile)	12 (6.7)	4 (2.7)	16 (4.5)
Obese (≥95th Percentile)	5 (2.8)	1(0.6)	6 (1.7)

## DISCUSSION

The present study was carried out to find out the morbidity patterns of the school children. School health survey gives a good chance to screen a large number of school children with minimum resources. In present study dental caries was found in 11.08%, followed by Tonsillitis 7.3%, Headache 5.06% and Eye problem 4.78%. The survey conducted by Shrestha et al. in Pokhara, found the prevalence of dental caries (41.5%), worm infestation (33.7%) and pediculosis (32.6%) in both sexes in six governmental primary school.^4^ The study conducted by Gokhale et al. found that the most common morbidities were dental caries (61.1%) followed by ear wax (38.1%), URTI (19%) and Ear discharge (13.5%).^5^ The study conducted by Mayavati et al. observed dental caries (65.1%), upper respiratory tract infections (38.2%), ear wax (29.9%) and myopia (10.0%) in urban school children.^6^ A study conducted in Madhya Pradesh, India by Shinde reported that 31.83% were having morbidity associated with dental carries followed by anemia (15.69%), vitamin A deficiency (6.25%), worm infestation (4.94%), scabies (4.06%), URI (3.77%), defective vision (0.58%), conjunctivitis (0.44%) and congenital malformation (0.15%).^7^ A study conducted by Bhandari et al. in Kavrepalanchowk district reported pediculosis 42 (26.2 %), dental caries 29 (18.1%), and waxy ear 27 (17.1 %) were three major morbidity in school children.^8^ Syed et al. conducted the study in Hyderabad, India found most common morbidities were Dental caries (56.24%), anemia (33.8%), worm infestation (48.25%) and Refractive error (11.22%).^9^ Kalyani et al. found most common morbidities were anemia (40%), Dental caries (61.6%), and pediculosis (40%). URT infection (32.8%) Refractive error (4%).^10^

These results were higher than that of the present study findings where dental caries was 11.08%, followed by Tonsillitis 7.3%, Headache 5.06% and Eye problem 4.78%. These differences may be due to the fact that the present study conducted in only a private school and improving health status as change with time period. The present study results were consistent with a study conducted by Pandey et al,^11^ Kausar et al,^12^ Shakya et al.^13^

In the World, the prevalence of malnutrition in term of Underweight, stunting and wasting are 27%, 31% and 10% respectively.^14^ In Nepal, more than 80% of the population lives in villages. Nepal Demographic and Health Survey, 2016 reported that 27% of children under 5 years were underweight, and 5% were severely underweight. The proportion of children who were underweight was greater in rural areas (31%) than urban areas (23%). Children of mothers with no education were more than twice as likely to be underweight as children whose mothers were educated up to SLC level. Underweight was inversely related to wealth quintile; 33% of children in the lowest wealth quintile were underweight, as compared with 12% of children in the highest quintile. 10% of children were wasted and 2% severely wasted. 36% of children were stunted, and 12% were severely stunted in Nepal (NDHS, 2017).^15^ A survey done by Shrestha et al found that the prevalence of wasting, stunting and malnutrition in the school children were 10.3%, 14.9% and 85% respectively with 31.8% of boys and 89.4% of girls students were malnourished.^4^ Study conducted by Bhandari et al found that 55.3% boys and 35.8% girls fell under 1st-degree malnutrition and 23.07% boys and 46.3% girls fell under 2nd-degree malnutrition, 7.2% girls fell under 3rd-degree malnutrition based on weight for age.^8^ The study conducted by Gokhale et al. found that the majority of school children (69.8%) were normal and 7.1% were severely underweight.^5^ The study carried out in Sullia town, South India by Amruth et al revealed the prevalence of underweight and stunting were 26.5% and 19.2% respectively.^16^ The nutritional status of government school children was comparatively poorer than private school children. Boys had a higher prevalence of malnutrition than girls. These findings were consistent with the present study where 24% of children were underweight and boys were more underweight than girls.

Gokhale et al. conducted the study in Maharashtra, India found 73% children had some morbidity. A study conducted in Madhya Pradesh, India reported morbidities were present in 58.72% of school children. The present study found that morbidities were present in 28.37% school children which were lower as compared to the other studies. This difference probably may be due to the present study conducted in grade I to X of urban area and in a private school where children were from educated and well up families. Overall present health status is also improving compared to past health status.

## CONCLUSIONS

Among different morbidities, dental caries was found more common. The health and nutritional status of school children were found to be satisfactory in this study compared to other studies. The present study emphasized the need for the initiation of oral health programs in schools. The study also reflects the importance of improving personal hygiene, prevention of disease like parasitic infection/infestation and improvement of their nutritional status. Today's children are the future of the country and the development of a country depends upon the development of children thus we should invest in school children.
